# Enhanced conversion efficiency in Si solar cells employing photoluminescent down-shifting CdSe/CdS core/shell quantum dots

**DOI:** 10.1038/s41598-017-14269-0

**Published:** 2017-10-26

**Authors:** R. Lopez-Delgado, Y. Zhou, A. Zazueta-Raynaud, H. Zhao, J. E. Pelayo, A. Vomiero, M. E. Álvarez-Ramos, F. Rosei, A. Ayon

**Affiliations:** 1 MEMS Research Lab, Department of Physics and Astronomy, University of Texas at San Antonio, San Antonio, TX 78249 USA; 2 Departamento de Física, Universidad de Sonora, Hermosillo, Son 83000 Mexico; 3 INRS Centre for Energy, Materials and Telecommunications, Varennes, QC J3X1P7 Canada; 4 Centro de Ciencias Exactas e Ingenierías, Universidad de Guadalajara, Guadalajara, Jal 44430 Mexico; 5 Luleå University of Technology, 971 87 Luleå, Sweden

## Abstract

Silicon solar cells have captured a large portion of the total market of photovoltaic devices mostly due to their relatively high efficiency. However, Silicon exhibits limitations in ultraviolet absorption because high-energy photons are absorbed at the surface of the solar cell, in the heavily doped region, and the photo-generated electron-hole pairs need to diffuse into the junction region, resulting in significant carrier recombination. One of the alternatives to improve the absorption range involves the use of down-shifting nano-structures able to interact with the aforementioned high energy photons. Here, as a proof of concept, we use downshifting CdSe/CdS quantum dots to improve the performance of a silicon solar cell. The incorporation of these nanostructures triggered improvements in the short circuit current density (J_sc_, from 32.5 to 37.0 mA/cm^2^). This improvement led to a ∼13% increase in the power conversion efficiency (PCE), from 12.0 to 13.5%. Our results demonstrate that the application of down-shifting materials is a viable strategy to improve the efficiency of Silicon solar cells with mass-compatible techniques that could serve to promote their widespread utilization.

## Introduction

Detailed studies on the global energy challenge indicate that photovoltaic (PV) technologies may contribute a fairly large fraction of future energy needs^1–3^. However, the mass production of PV solar cells involves a substantial consumption of natural resources.

It is possible to identify three types of solar cells including i) first generation solar cells mainly based on silicon substrates; ii) second generation solar cells based on thin films of amorphous silicon, copper indium gallium selenide (CIGS) and CdTe; iii) third generation solar cells using organic dye molecules, polymers or semiconducting inorganic nanocrystals or hybrid materials^4,5^. Very recently, among third generation solar cells, hybrid perovskite based PV devices were observed to exhibit power conversion efficiencies (PCE) exceeding 20%, maintaining 90% of their initial performance after 500 hours under one sun illumination (100 mW/cm^2^)^6^.

Current commercial solar cells may be classified into four main technologies: silicon wafer-based, thin film Silicon, cadmium telluride (CdTe) and CuIn_X_Ga_1-X_Se_2_ (CIGS). In each case, there are specific limitations, due for example to existing supplies for one or more of its components^7–9^. In the case of CdTe technology, the main limitation is related to the limited Tellurium reserves. In CIGS technology, the availability of Indium, Gallium and Selenium limit the potential market fraction of these solar cells. Currently, silicon based solar cells still dominate the PV market (~85%), also due to existing fabrication and technology infrastructure. One of the main limitations of Silicon-based technologies arise from the amount and cost of the typical electrodes employed (namely silver-based contacts). This problem could be addressed by using abundant and affordable materials like aluminum-based metallization^10^, ensuring that Si solar cells remain the most widespread PV technology in the market. Presently, research on Si solar cells focuses on improving the PCE by fabricating heterojunction tandem solar cells,^11^ enhancing the absorption efficiency of thin-film solar cells through various photonic patterns,^12–14^ and by Multiple-Exciton Generation^15,16^ or space separating quantum cutting^17^ etc., which represent promising opportunities to increase the PV contribution to the energy portfolio.

Recent efforts in this direction have focused on different methods, including surface nanostructuring, antireflection coatings and surface passivation schemes, among others^18–21^. One of the main challenges is the efficient exploitation of photons absorbed by Silicon to efficiently produce electric power^22,23^. The wavelength distribution of the solar spectrum extends from the infrared (2500 nm) to the ultraviolet (UV, 280 nm) but only a fraction is absorbed by materials typically employed in solar cells^24,25^. The underlying reasons are that in principle only photons with energy greater than the bandgap can be absorbed, and at the same time, photons with energies much larger than the bandgap do not produce equivalent electric power, but only contribute to thermalization within the material, leading to inevitable energy losses^24^, or are absorbed at the highly doped surface region, where excitons rapidly recombine into light or heat before separation. These losses can be drastically reduced by using luminescent materials, which can either induce charge photogeneration from absorption of low-energy photons through up-conversion, or limit thermalization charge losses via the down-shifting of high-energy photons before charge photogeneration.

The absorption of low energy photons (near and below the bandgap) can be promoted by using nanoscale systems which exhibit up-conversion properties^26–28^. This effect consists in the simultaneous absorption of at least two low energy photons (with energy below the energy gap) and the emission of one higher energy photon, which can be absorbed by the photovoltaic material employed. On the other hand, when photons are too energetic and only contribute to thermalization within the device, down-shifting materials^28–32^ can absorb a single high-energy photon, typically in the UV range, and re-emit one or two lower energy photons, typically in the visible and infrared, in the optimum range of absorption of the material constituting the PV device, increasing the PCE.

Here we report the synthesis and characterization of photoluminescent, down-shifting, core-shell CdSe/CdS quantum dots (QDs) that absorb in the UV range of the solar spectrum and emit photons with wavelengths centered ~625 nm, which is well suited for Silicon absorption and electron-hole pair generation. We demonstrate a model system by deploying these QDs on the window side of single-crystal-Silicon solar cells. The incorporation of the CdSe/CdS QDs boosts the short circuit current density (J_sc_), respectively, with an increase in the PCE from 12.0 to 13.5%.

## Results

**Figure 1:**
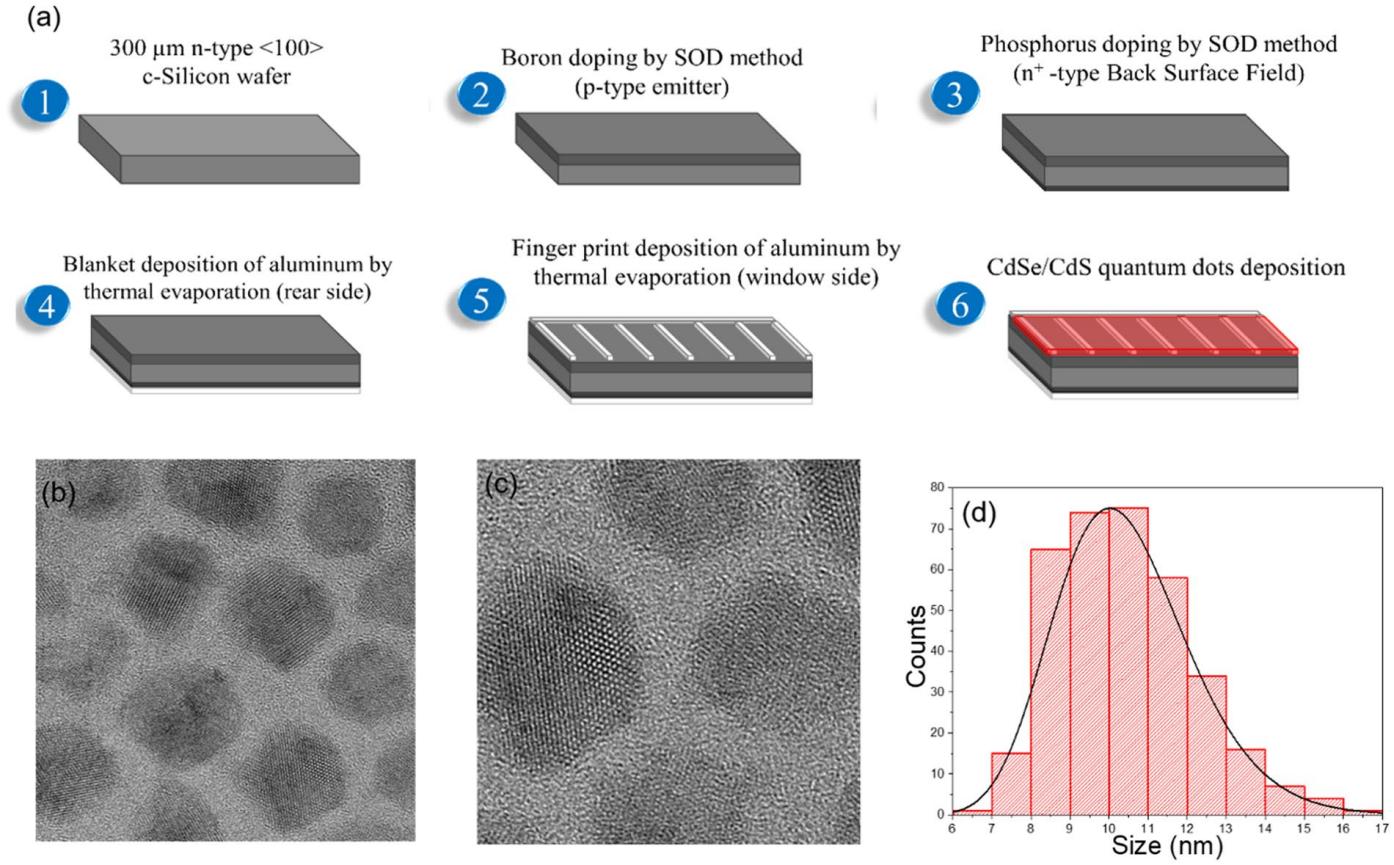
(**a**) Flowchart of the fabrication of c-Silicon solar cell and CdSe/CdS-QDs deposition, (**b**) and (**c**) TEM and HR-TEM of the QDs and (**d**) QDs size distribution, obtained from TEM analysis.

CdSe QDs were synthesized by using the hot injection approach^30^. CdS shell on CdSe QDs was grown via a successive ionic layer absorption and reaction (SILAR) approach following the procedure described in Ghosh *et al*.^33^. Figure 1a shows the flowchart of the fabrication and deposition process. Detailed information for the fabrication of the solar cells are included in the Methods Section. The starting CdSe QDs have a core size of 3.27 nm in diameter with a uniform size distribution (standard deviation σ < 10%). After the growth of 13 monolayers via a SILAR approach, the final diameter was around 10.44 nm for the CdSe/13CdS QDs as shown in Fig. 1d (shell thickness ~3.6 calculated based on the method in ref.^33^). The crystal lattice is clearly visible in the high-resolution transmission electron microscope (HRTEM) image (Fig. 1b-c).

**Figure 2:**
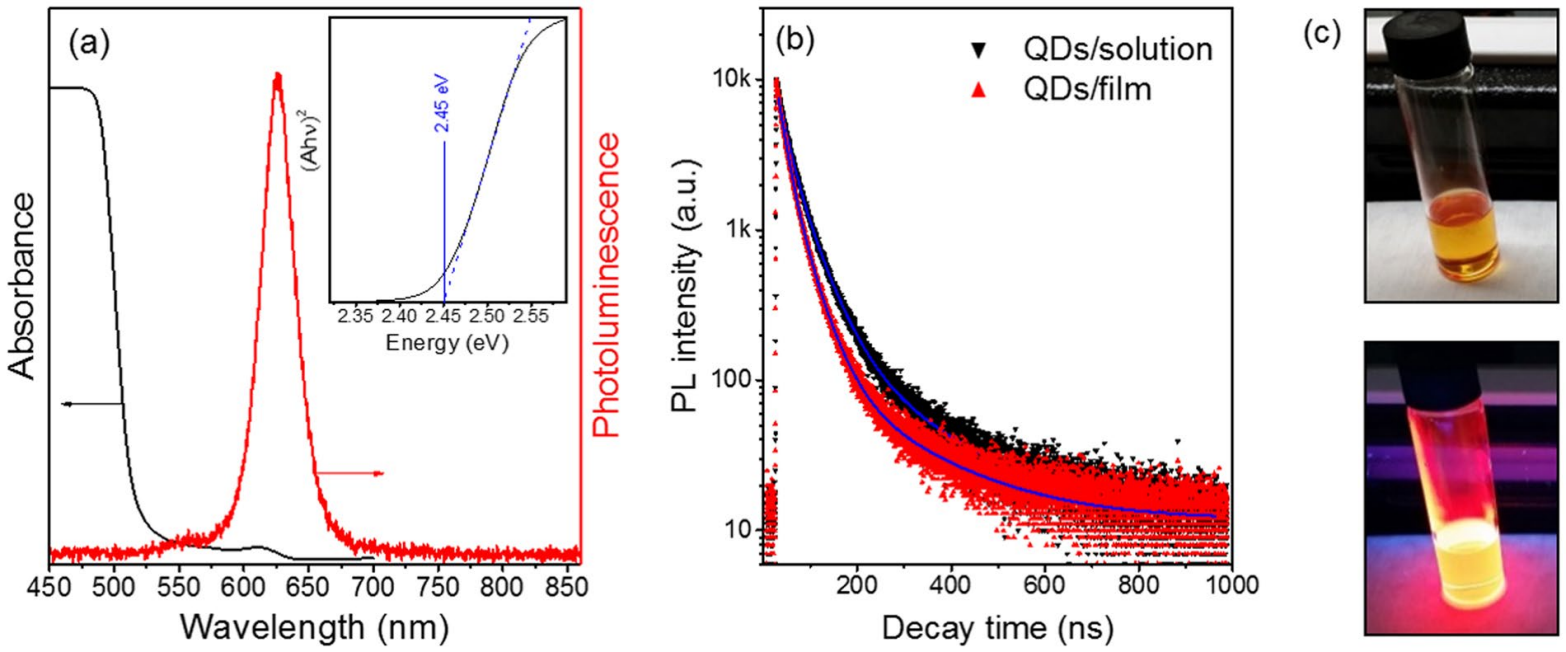
(**a**) Absorption (black) and PL (red) spectrum of CdSe/CdS QDs. Excitation wavelength for PL: λ_ex_ = 420 nm. Inset in (**a**): Tauc’s plot for determining the energy gap, resulting in an *E*_g_ = 2.45 eV for the analyzed QDs. (**b**) PL decay for QDs in solution and in thin film. (**c**) Images of QD solution under visible (top) and UV light (bottom).

The optical properties of core/shell QDs are shown in Fig. 2 (including images of samples in solution under visible and UV light). CdSe/CdS QDs show the absorption spectrum ranging from below 450 to 625 nm. The absorption spectrum of CdSe/CdS QDs (Fig. 2a) is dominated by the optical features of the CdS shell (<500 nm), due to the larger shell volume compared to the core, even if the CdSe core absorption is still present (weak absorption in the region 500–625 nm). The photoluminescence (PL) maximum of the core/shell QDs is located at 625 nm with a narrow peak width due to the monodisperse size distribution of starting CdSe core and uniform CdS shell thickness (Fig. 1d). The large Stokes shift in the CdSe/CdS QDs (the red shift of the emission spectra with respect to absorption spectra, ∼120 nm in the present case) can largely decrease the self-absorption of QDs after excitation, making them good candidates as light down-converters for Si solar cells^34,35^. Compared to a Si nanocrystal-based luminescent down-shifting layer^32^, CdSe/CdS QDs exhibit a stronger absorption in the visible range 400–500 nm and a larger absorption coefficient. Additionally, the synthesis of colloidal low-cost CdSe/CdS QDs is simpler and suitable for production in large quantities^33,36^. Although CdSe/CdS QDs may be considered relatively more toxic compared to Si nanocrystals, only low-quantities are used in thin films, and with a proper encapsulation scheme the CdSe/CdS QDs will not leak into the environment.

Using Tauc’s Law and the absorption spectrum, we can estimate the bandgap of the synthetized CdSe/CdS QDs. In accordance to this law, for a direct bandgap material:

1 $${B}_{1}{(Ah\nu )}^{2}+{E}_{g}=h\nu $$

where *A* is the experimental absorbance, proportional to the absorption coefficient, *h* is Planck’s constant, *v* is the photon frequency, *E*_*g*_ is the bandgap energy and *B*_1_ is a constant. The calculated *E*_*g*_ value employing this method was 2.45 eV (see inset in Fig. 2a).

We compared the PL lifetime of QDs in solution and after formation of the thin film through spin coating (Fig. 2b), to confirm the presence of long-lived excited states in thin film, with limited effect of QD-QD interactions, which increases non-radiative exciton recombination, inducing photon loss. Transient PL intensity is well fitted for both solution and thin film by a three-exponential lifetime. We calculated the average lifetime *τ*_av_ according to the following equation:

2 $${\tau }_{{\rm{av}}}=\sum {\tau }_{i}{{\rm{B}}}_{i}$$

Where *τ*_i_ are the three lifetime components of the fitting and B_i_ (i = 1, 2, 3) are their relative intensities. Thin film QDs exhibit an average lifetime of (46 ± 2) ns, slightly lower than the QDs in solution (*τ*_av_ = (59 ± 3) ns), yet still demonstrating a low QD-QD interaction, which could hinder its application as a down-shifting layer.

Based on the results of QD absorption, PL emission and PL lifetime in thin films, the QD film presents the desired optical properties to be applied as an efficient down-shifting layer for crystalline silicon (c-Si) solar cells.

**Figure 3:**
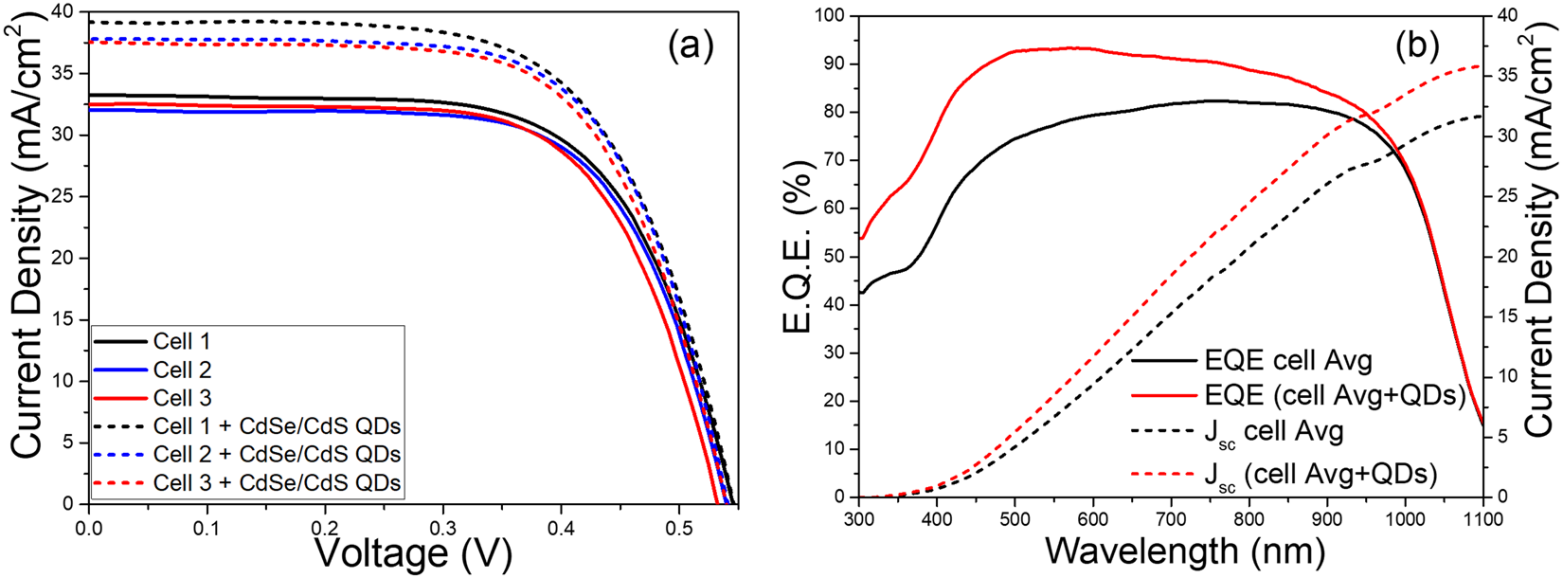
(**a**) Current-Voltage characteristics (solid line: without QDs; dashed line: with QDs). (**b** left) measured EQE of the solar cells. (b right) Calculated short circuit current density from EQE data, according to Eq. 3 (black: without QDs; red: with QDs). All the samples were characterized by the same method in order to compare the performance of the device with and without the influence of the CdSe/CdS QDs.

In this study, we fabricated c-Si solar cells as a model system, serving as a prototype to investigate the effects of down-shifting QDs. The current density-voltage (J-V) curve of the finished solar cell was recorded using an Oriel Sol2A solar simulator under AM1.5 G illumination (100 mW/cm^2^) at standard testing conditions. External quantum efficiency (EQE) characterization was performed with a Newport External Quantum Efficiency Measurement System. Three solar cells were considered for each condition (with/without QDs) to provide quantitative information on the reproducibility of the results and on the accuracy of the measurement. The J-V curves for the three samples before and after the deposition of the QD film are reported in Fig. 3a. The average solar cell parameters are reported in Table 1. As shown by the J-V curves, the procedure for solar cell fabrication is highly reproducible, giving very similar values of the functional parameters for the three samples. The application of the QDs in c-Si solar cells leads to increases in the short circuit current density (J_sc_). An improvement was observed in all the solar cells after the deposition of QDs, where a noticeable increase in the J_sc_ from an average value of (32.5 ± 0.6) to an average value of (37.0 ± 0.6) mA/cm^2^ was observed, leading to an average improvement of the PCE from (12.0 ± 0.2)% to (13.5 ± 0.2)% for an overall PCE improvement of 12.7%.

**Table 1:** c-Silicon solar cell performance parameters before and after application of CdSe/CdS QDs.

Sample	Functional parameters				
	V_oc_ (mV)	J_sc_ (mA/cm^2^) (measured)	J_sc_ (mA/cm^2^) (from EQE)	FF (%)	PCE (%)
Solar cell set without QDs	543.4 ± 6.8	32.5 ± 0.6	32	68.0 ± 0.9	12.0 ± 0.2
Solar cell set with QDs	545.9 ± 3.4	37.0 ± 0.6	36	67.0 ± 1.0	13.5 ± 0.2

The values are an average of three nominally identical samples, whose current density-voltage and EQE curves are reported in Fig. 3.

The EQE was measured to study the spectral response of the fabricated c-Si solar cells with and without the deployed CdSe/CdS QD films as shown in Fig. 3b. The EQE response improved in a wavelength segment extending from the UV region to the visible, with the maximum increase (more than 10%) between 350 and 630 nm. This improvement is considered to be responsible for the increase in photocurrent density.

**Figure 4:**
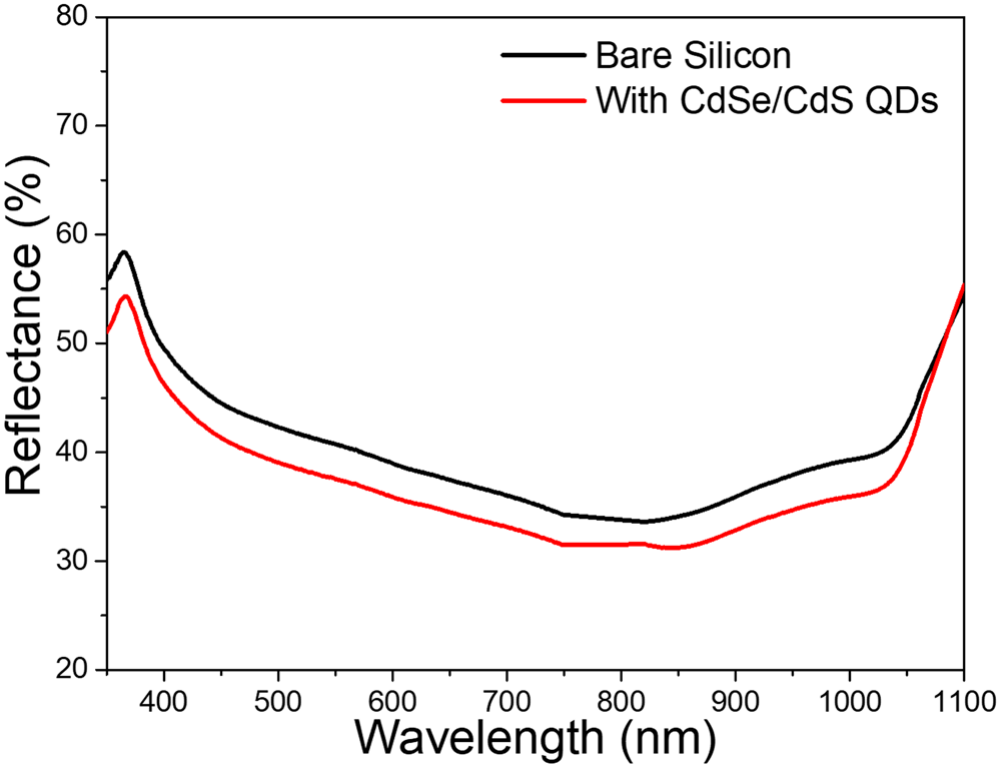
Reflectance from the surface of the c-Silicon solar cell before and after deposition of the QD film. QD film has a clear (though weak) antireflection effect in the whole range of interest for photoconversion.

To verify that the increased photocurrent density originates from the downshifting action of the QDs, reflectivity measurements were collected on the surface of the solar cells before and after the deposition of the QD film (Fig. 4). The presence of the QDs has a beneficial effect in decreasing reflectivity in the full range (300–1050 nm) and is rather uniform in the analyzed range. The increased EQE values in the region 350–630 nm can be attributed both to the reduced reflectivity and the down shifting effects of the QDs employed. The increase in EQE does not follow the trend of reflectance (which is almost constant in all the spectral region), but it is more pronounced in the absorption region of the QDs. This could be explained from the fact that reflectance spectroscopy considers only photons that are either absorbed or reflected (under the assumption of no-transmission) but it does not take into consideration the probability of absorbed photons to contribute to electron-hole pair generation in a solar cell. Therefore, since the QDs employed exhibit down shifting properties, it can be ascertained that photons in the previously mentioned range are absorbed and downshifted to a wavelength more favorable for photocurrent generation, leading to an enhanced value of the measured EQE. The antireflection properties of the QD film contribute to the increase in EQE.

On the other hand, the increase observed in the spectral region 630–1000 nm can be attributed to the change in the reflectance of the device due to the presence of the antireflection properties of the CdSe/CdS QDs layer.

In addition, EQE measurements were employed to provide an independent corroboration of the J_sc_ values collected with the solar simulator, the short circuit current density was extracted from the EQE spectra by using the relation:

3 $${J}_{sc}=q{\int }^{}{b}_{s}(E)EQE(E)dE$$

where *q* is the electron charge and *b*_*s*_(*E*) is the solar incident spectral photon flux density. Evidently since EQE is employed to provide an independent verification of the measured values of the short circuit current density (equation 3), it is, therefore, an indicator of the consistency of the collected values.

As shown in Fig. 3b and Table 1, the calculated Jsc from EQE spectra are 32 mA/cm^2^ (the measured Jsc: 32.5 ± 0.6 mA/cm^2^) for solar cell without QDs and 36 mA/cm^2^ for solar cell with QDs (the measured Jsc: 37 ± 0.6 mA/cm^2^), respectively. A good agreement between the calculated Jsc and the Jsc measured is found from the J-V experimental curve. In all cases, the current density values calculated using the aforementioned equation are in agreement with those collected during J-V characterization using the solar simulator, confirming the improvement of the generated photocurrent.

**Figure 5:**
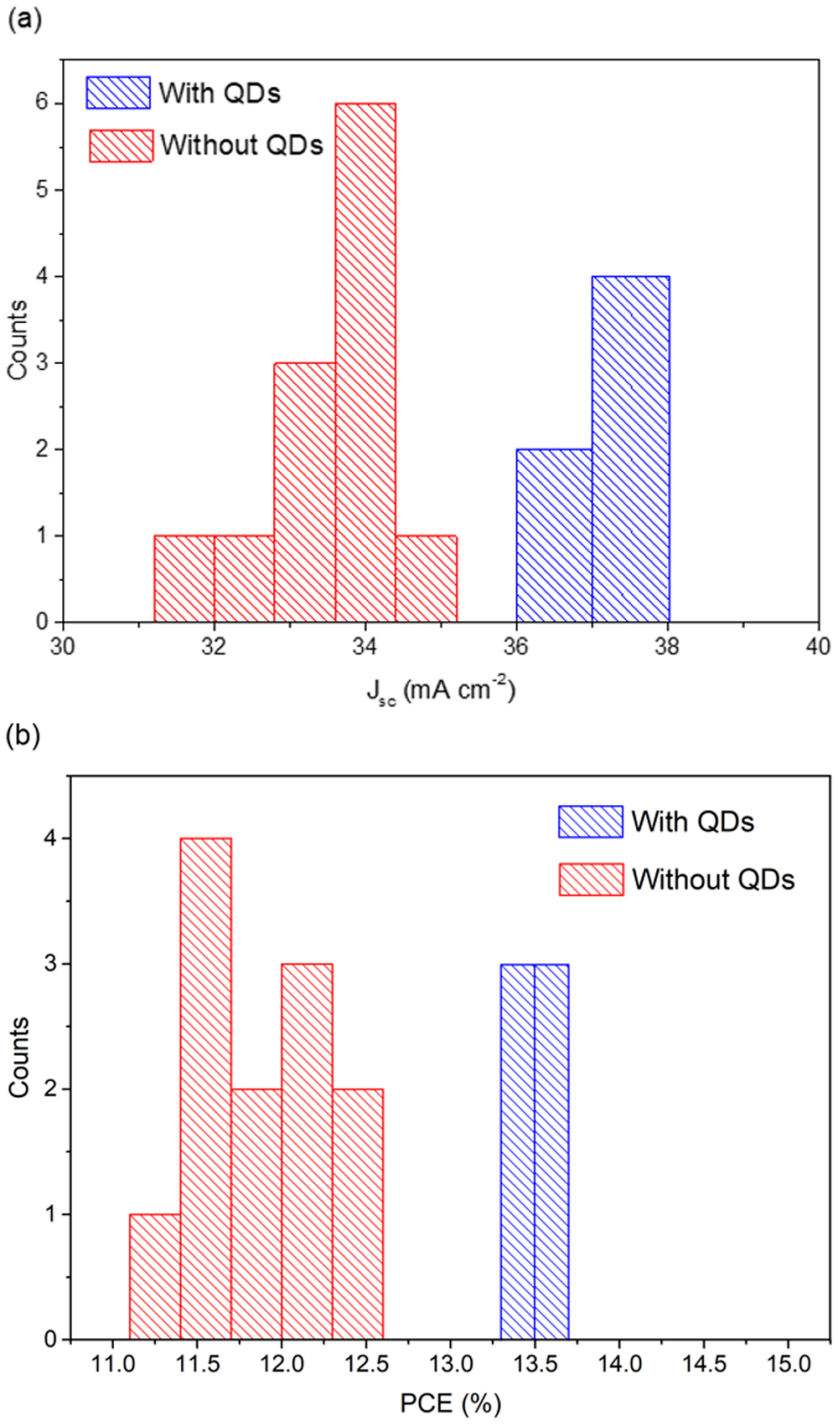
Histogram of distribution of short circuit current density (**a**) and photoconversion efficiency (**b**) for two different populations of Si solar cells with and without QDs. 12 solar cells are considered in the population without QD treatment and six solar cells were treated using the downconverting QDs.

To validate the statistical relevance of the data, we measured a set of 12 silicon solar cells without QD addition, whose J_sc_ and PCE are reported as histograms in Fig. 5 together with the values from six solar cells treated with QDs. We obtained the following average values and standard deviations: J_sc_ = (33.5 ± 0.8) mA/cm^2^ and PCE = (11.9 ± 0.3)% for cells without QDs; J_sc_ = (37.2 ± 0.5) mA/cm^2^ and PCE=(13.5 ± 0.1)% for cells after QD addition. These results demonstrate no overlap within the confidence interval between the two distributions of the cells, confirming the validity of our hypothesis and the effectiveness of the QDs in boosting the functional properties of Si solar cells. The investigated solar cells, based on Si, exhibit a degree of reproducibility much larger than other types of solar cells, like, for instance, perovskite-based solar cells, for which the standard deviation on PCE is much larger than those reported herein, as clearly demonstrated in ref.^37^.

### Conclusions and Perspectives

In conclusion, we demonstrate a prototype system in which the performance of a c-Si solar cell is improved by introducing down-shifting CdSe/CdS QD thin films. The observations indicate that the incorporation of CdSe/CdS QDs as down-shifting material on top of a c-Si solar cell improves photocurrent generation, resulting in an increase in the short circuit current density, as well as the EQE. Even though the FF was observed to slightly decrease in all cases, the overall PCE achieved after incorporating CdSe/CdS-QDs increased from 12.0% to 13.5% (a relative improvement of 12.7%). The strategy described herein is robust, cost-effective and promising towards improving the efficiency of existing Si-based solar cells, which could help increase the market share of solar technologies. It is anticipated that when an antireflective coating is employed, the thickness may have to be adjusted to permit the incorporation of QDs on the window side of the solar cell to preserve both anti-reflecting as well as the energy downshifting properties. Future strategies to increases the PCE of silicon solar cells include doping with Pb the CdS shell^33^ to increase the absorption range of CdSe/CdS QDs and improving the quantum yield of QDs.

## Methods

### Materials

Sulfur (100%), Oleylamine (OLA, technical grade 70%), Cadmium Oxide (CdO, 99%), Oleic Acid (OA), Rhodamine 6 G, 1-Octadecene (ODE), Selenium Pellet (≥99.99%), Trioctyl Phosphine Oxide (TOPO), Trioctyl Phosphine (TOP, 97%), Hexane, Toluene, Ethanol, Hydrogen Peroxide 35% (H_2_O_2_), Ammonium Hydroxide 30% (NH_4_OH), Hydrochloric Acid 37% (HCl) and Hydrofluoric Acid 49% (HF) were used as purchased.

### Synthesis of QDs

TOPO (1 g) and^33,36^ Cd-oleate (0.38 mmol, 1 mL) in 8 mL of ODE were purged by N_2_ at room temperature for 30 min. The reaction system was evacuated for 30 min at 100 °C, then the temperature was increased to 300 °C. A mixture of TOP-Se (4 mmol, 4 mL), 3 mL of OLA and 1 mL of ODE was quickly injected into the Cd-oleate suspension under vigorous stirring. To clean the core CdSe QDs ethanol was added, then the suspension was centrifuged (6000 r.p.m.) for 5 min and the supernatant was removed. The core CdSe QDs were then dispersed in toluene. After the core was synthetized, the shell was deposited through a Cd and S layer by SILAR. For this purpose, OLA (5 mL), ODE (5 mL) and CdSe QDs (∼2 × 10^−7^ mol in hexane) were degassed at 110 °C for 30 min. Then the temperature was raised to 240 °C with stirring. Cd-oleate dispersed in ODE (0.25 mL, 0.2 M) was added dropwise and the mixture was allowed to react for 2.5 h, followed by dropwise addition of 0.2 M sulfur in ODE with same volume and a reaction time of 1 h. The shell was further annealed for 10 min. All subsequent shells were annealed at 240 °C for ~10 min. The reaction was cooled to room temperature using ice water (0 °C). Ethanol was added, the suspension was centrifuged (6000 r.p.m.) and the supernatant was removed. The QDs were then dispersed in toluene for further characterization.

### Fabrication of single crystal Silicon solar cells

Single crystal Silicon solar cells were fabricated employing 4-inch, n-type, <100> Silicon wafers with a resistivity of 10–20 Ω-cm employing a spin on dopant technique (SOD) that has been described elsewhere^38^. The Silicon samples were cleaned using an extended 4-step RCA cleaning process consisting of an extra oxide layer removal. To this end, the samples were immersed in a solution of de-ionized water (DI-water), H_2_O_2_ (35%) and NH_4_OH (30%) with a volume ratio of 5:1:1 at 80 °C for 10 min to remove organic residues. The second step consisted in the removal of the native oxide layer by a short immersion of the sample in a solution of DI-water and HF (49%) in a ratio 50:1 for 60 s at room temperature. The wafers were then immersed in a solution of DI-water, H_2_O_2_ and HCl (37%) in a volume ratio of 5:1:1 at 80 °C for 10 min to remove any remaining metallic ion contaminants. Finally, the samples were cleaned in the previously prepared diluted HF solution for 60 s at room temperature to remove the oxide layer created in the third step. After each step, the samples were rinsed with DI-water and dried with a stream of nitrogen. To create the p-type emitter layer and the n+back surface field (BSF) for charge collection and transport, Boron and Phosphorous SOD solutions were prepared by the sol-gel method^38^. The Boron (SOD) solution was deposited on the window side of the Silicon sample and the Phosphorous (SOD) on the opposite side. Then, the samples were annealed at 120 °C for 10 min to remove the organic solvents. Subsequently, the samples were annealed at 1000 °C for 10 min to diffuse the dopants and create the p–n junction and the BSF. The parasitic silicate glass layer, typically formed when employing the previously described doping method, was removed by immersing the samples in a dilute HF aqueous solution for 120 s. The sheet resistivities of the samples were measured using a four-point probe tool. To make the electrical contacts, 200 nm of aluminum were deposited on each side of the device by a thermal evaporation technique. A shadow mask was used on the window side to produce a pattern of finger electrodes, while a blanket deposition was performed on the back side. Finally, the samples were annealed at 585 °C for 10 min to promote the formation of an ohmic Al/Si contact. A set of three solar cells was fabricated and characterized, before and after coverage of the surface with CdSe/CdS-QDs. To this end, 200 μL of CdSe/CdS-QDs in toluene was dispersed via spin coating with an angular velocity of 5000 r.p.m for 60 s.

### Characterization

Structural characterization of the QDs was carried out with a JEOL 2010-F TEM working at 200 kV. UV/Vis absorption spectroscopy of the synthesized CdSe/CdS-QDs was performed employing a Varian Cary 5000 UV-Vis spectrometer. The PL response was recorded using an Ocean Optics Flame-S-UV-Vis spectrometer, using an excitation wavelength of 420 nm. The PL lifetime of the QDs in film was measured using a pulsed laser diode of 405 nm and TCSPC Model in the Fluorolog®−3 system. The J-V curve of the finished solar cell was recorded using an Oriel Sol2A solar simulator under AM1.5 G illumination (100 mW/cm^2^) at standard testing conditions. EQE characterization was performed with a Newport External Quantum Efficiency Measurement System. Reflectivity measurements were collected on the surface of the solar cells before and after the deposition of the QD film (Varian Cary 5000 UV-Vis spectrometer coupled with an integrating sphere).
